# Integrative Computational Analysis of *TP53* Exon 5–6 Mutations in Oral Cavity, Prostate, and Breast Cancers in a Senegalese Population

**DOI:** 10.3390/genes17020245

**Published:** 2026-02-20

**Authors:** Mouhamed Mbaye, Fatimata Mbaye, Mbacke Sembene

**Affiliations:** 1 Pharmaceutical Biophysics Laboratory, Department of Pharmacy, Faculty of Medicine, Pharmacy and Odontology, Cheikh Anta Diop University, Dakar BP. 5005, Senegal; mouhamed19.mbaye@ucad.edu.sn; 2 Genomics Laboratory, Department of Animal Biology, Faculty of Science and Technology, Cheikh Anta Diop University, Dakar BP. 5005, Senegal; mbacke.sembene@ucad.edu.sn

**Keywords:** *TP53*, cancer, oral cavity, prostate, breast, Senegal, mutation, bioinformatics

## Abstract

**Background/Objectives**: The tumor suppressor gene *TP53* is one of the most frequently mutated genes in human cancers, with alterations predominantly affecting its DNA-binding domain (DBD). However, the mutational landscape and functional consequences of *TP53* variants remain poorly characterized in African populations. This study aimed to characterize mutations in exons 5–6 of *TP53* in oral cavity cancer (OCC), prostate cancer (PC), and breast cancer (BC) in a Senegalese population, and to assess their structural effects, functional consequences, and impact on protein–protein interactions with BCL-2. **Methods**: Seventy-eight archived tumor DNA samples from Senegalese patients with OCC, PC, and BC were analyzed. Variants were annotated using COSMIC and dbSNP databases. Functional impact was evaluated with PolyPhen-2. Structural stability changes (ΔΔG) were predicted using FoldX, conformational dynamics ($\Delta \Delta S_{vib}$) were assessed with ENCoM, and effects on the p53–BCL-2 interaction were analyzed using DDMut-PPI. Statistical analyses were also performed. **Results**: BC exhibited the highest *TP53* mutation frequency, whereas OCC showed greater mutational diversity. Exon-level analysis revealed a significant enrichment of exon 6 mutations in BC. Structural analyses indicated that exon 5 mutations across all cancers and mutations in OCC were predominantly destabilizing and associated with loss-of-function effects. In contrast, recurrent exon 6 mutations in PC and BC, particularly V217L and V218M, were predicted to stabilize the p53 structure. Conformational dynamics differences between exons were significant only in PC. All analyzed mutations were predicted to stabilize the p53–BCL-2 interaction. **Conclusions**: This integrative in silico study identified cancer and exon-specific *TP53* mutation patterns in a Senegalese population, highlighting exon 6 as a context-dependent hotspot with potential oncogenic implication in PC and BC. Despite its computational nature, the study provides valuable insights that merit further investigation.

## 1. Introduction

Although not all cancers are initiated by detectable gene mutations, cancer development generally involves a complex interplay of genetic and epigenetic alterations, environmental exposures, and cellular stress [1]. Among the genes most frequently affected, *TP53* stands out as the single most mutated gene in human cancers, with alterations identified in approximately 50% of solid tumors [2]. *TP53* encodes the tumor suppressor protein p53, a transcription factor central to maintaining genomic integrity through its roles in DNA repair, cell cycle arrest, senescence, and apoptosis [3]. Mutations affecting the DNA-binding domain (DBD), which spans exons 5 to 8, are particularly disruptive, as this region is essential for sequence-specific DNA recognition and transcriptional regulation [4]. In addition to its canonical functions, p53 interacts with several regulatory pathways, including apoptotic mediators such as BCL-2 [5]. Under physiological conditions, activated p53 represses *BCL2* expression to promote apoptosis, helping eliminate damaged or potentially oncogenic cells. Alterations in *TP53* can therefore compromise not only DNA damage responses but also apoptotic signaling, contributing to tumor progression and therapy resistance [6].

The distribution and functional impact of *TP53* mutations vary extensively across cancer types, geographical regions, and populations [7,8]. Factors such as genetic background, lifestyle, and environmental exposure shape the mutational landscape of cancers [9]. This heterogeneity involves both somatic and germline variants. While the present study focuses on somatic variants identified in tumor tissue, it is important to note that inherited polymorphisms can also modulate cancer risk. For instance, the germline Pro47Ser (rs1800371) polymorphism, enriched in individuals of African ancestry, has been associated with reduced p53 tumor-suppressive activity and increased susceptibility to breast cancer (BC) [10]. Despite this diversity, African populations remain underrepresented in cancer genomics research, resulting in limited knowledge about mutation patterns and their biological implications [11].

In Senegal, oral cavity cancer (OCC), prostate cancer (PC), and BC represent growing public health concerns. Recent clinical and epidemiological data have documented a notable rise in their incidence, yet molecular studies focusing on *TP53* remain scarce [12,13,14]. Understanding the mutational spectrum of *TP53* and its potential functional consequences is essential for advancing molecular oncology in these populations.

The present study aims to characterize mutations occurring in exons 5 and 6 of *TP53* in Senegalese patients diagnosed with OCC, PC, and BC by using a comprehensive computational framework. Through the integration of sequence analysis, pathogenicity prediction, structural stability assessment, molecular dynamics evaluation, and protein–protein interaction (PPI) modeling, we sought to gain insight into how these variants may influence p53 structure and function.

## 2. Materials and Methods

### 2.1. Ethical Considerations

This study involved the retrospective analysis of anonymized DNA sequences obtained from archived diagnostic materials in the Genomics Laboratory of the Faculty of Science and Technology at Cheikh Anta Diop University. No patient-identifying information, clinical metadata, or demographic variables were used. Because all samples were pre-existing, fully anonymized, and analyzed exclusively for secondary research purposes, formal institutional ethics approval was not required, in accordance with international guidelines for retrospective studies using de-identified biological material.

### 2.2. Patients and Sampling

A total of 78 anonymized DNA samples corresponding to Senegalese patients diagnosed with OCC (*n* = 40), PC (*n* = 18), and BC (*n* = 20) were retrospectively retrieved from institutional diagnostic archives. Patients with OCC were managed at the Maxillofacial Surgery Department of Hôpital Aristide Le Dantec (HALD), patients with PC at the Department of Anatomical Pathology of Hôpital Régional El Hadji Ahmadou Sakhir Ndiéguène de Thiès, and patients with BC at the Institut Joliot-Curie of HALD. DNA samples were originally collected as part of routine diagnostic workflows and subsequently processed and archived at the Genomics Laboratory of the Faculty of Science and Technology, Cheikh Anta Diop University (UCAD). Sample collection spanned the period from 2018 to 2020.

DNA extraction was previously performed using the Zymo Research Kit (Zymo Research Corp., Irvine, CA, USA) following the manufacturer’s instructions. Archived DNA samples had previously undergone PCR amplification of exons 5 and 6 using the following primer pair: Forward: 5′-GTTTCTTTGCTGCCGTCTTC-3′, Reverse: 5′-CTTAACCCCTCCTCCCAGAG-3′. Amplicons were verified on 2% agarose gel with a 100 bp SmartLadder and sequenced using Sanger sequencing at Macrogen Europe B.V. (Amsterdam, The Netherlands).

Exons 5 and 6 of *TP53* were selected because they encode the core of the DBD, which harbors multiple mutational hotspots critical for p53 function. These exons were also the only regions consistently amplified and archived in the participating diagnostic laboratories. Although exons 7 and 8 also belong to the DBD and include known hotspots, they were unavailable in the archived sequencing datasets. Consequently, this study focused on exons 5–6 and acknowledges this limitation. Future work will extend the analysis to exons 5–8.

### 2.3. Sanger Quality Control and Variant Validation

Because Sanger sequencing does not generate depth-of-coverage metrics as in NGS, quality control relied on electropherogram inspection and Mutation Surveyor v5.2 default filtering parameters, including peak morphology and symmetry, peak-to-noise ratio thresholds, automated artifact removal (dye blobs, shoulder peaks, baseline noise), and forward–reverse signal concordance. Mutation Surveyor’s numerical mutation score (NM score) was used to assess confidence. Variants with NM score > 20 were accepted as high confidence following the threshold recommended by the software manufacturer, where NM scores above 20 indicate strong mutation signal reliability and minimal likelihood of artifact. Ambiguous chromatogram regions were manually inspected, and any sequence with unresolved noise or poor alignment quality was excluded. Rare variants were validated through manual chromatogram verification, forward–reverse comparison and re-alignment using Biopython’s Bio.Align module (Biopython v1.81) and ClustalW2 v2.1 to exclude alignment artifacts.

Somatic status was inferred through a multi-step annotation strategy. First, all variants listed in COSMIC v97 were classified as somatic. Then, variants present in dbSNP build 155 with documented non-zero population allele frequencies were considered germline polymorphisms and excluded from further analysis. Finally, variants that appeared in dbSNP without allele-frequency information, as well as those absent from both COSMIC and dbSNP, were retained as somatic-like when they occurred within established *TP53* hotspot regions or exhibited substitution patterns characteristic of tumor-associated mutations such as C>T transitions at CpG dinucleotides.

### 2.4. Computational Analyzes

#### 2.4.1. Functional Impact Assessment

Single nucleotide missense variants (mSNVs) were evaluated using Polymorphism Phenotyping-2 (PolyPhen-2) v2.2.2, which classifies variants as probably damaging, possibly damaging, or benign [15]. Predictions were based on the reference sequence NP_000537.3.

#### 2.4.2. Impact on Structural Stability

The Gibbs free energy (ΔG) of mutant p53 was estimated using FoldX 5.0 via the Pyfolfx library to assess the effects of mutations on p53 structural stability [16,17]. ΔG measures protein stability, determining whether a given structure is thermodynamically favorable or unfavorable. FoldX 5.0 calculates ΔG by decomposing energy contributions from various interaction types within the protein, including Van der Waals forces, hydrophobic interactions, hydrogen bonds, electrostatic interactions, backbone and sidechain entropies, and steric clashes. For each mutation, FoldX 5.0 calculates the difference in Gibbs free energy (ΔΔG) by subtracting the ΔG of the wild-type protein from that of the mutant: $\Delta \Delta G=\;\Delta G_{mut}-\;\Delta G_{wt}$. By convention, the mutant protein is significantly unstable if ΔΔG > 1 kcal·mol^−1^, neutral if between −1 and 1 kcal·mol^−1^, significantly stable if ΔΔG < −1 kcal·mol^−1^ [18]. The crystallographic structure 2FEJ from the Protein Data Bank (PDB) was used for this evaluation, and the “RepairPDB” function was applied before mutation modeling.

#### 2.4.3. Impact on Molecular Dynamics

Mutation-induced changes in vibrational entropy ($\Delta S_{vib}$) and flexibility were estimated using DynaMut v1.3 [19]. It employs normal mode analysis (NMA) using the Elastic Network Contact Model (ENCoM) [20]. ENCoM models the protein as an elastic network model where residues represent nodes and interactions are modeled as springs. The difference in vibrational entropy ($\Delta \Delta S_{vib}$) is obtained by subtracting the ΔS_vib of the wild-type protein from that of the mutant: $\Delta \Delta S_{vib}\;=\;\Delta S_{vib}^{mut}-\;\Delta S_{vib}^{wt}$. ENCoM $\Delta \Delta S_{vib}$ values were classified as stabilizing ($\Delta \Delta S_{vib}$ < −0.1 kcal·mol^−1^·K^−1^), neutral (−0.1 ≤ $\Delta \Delta S_{vib}$ ≤ +0.1 kcal·mol^−1^·K^−1^), or destabilizing ($\Delta \Delta S_{vib}$ > +0.1 kcal·mol^−1^·K^−1^) using a ±0.1 kcal^−1^·mol^−1^ threshold commonly applied to account for model precision and to avoid overinterpreting minimally small entropic fluctuations. The same crystallographic structure 2FEJ was used as a template.

#### 2.4.4. Impact on p53-BCL2 Protein-Protein Interactions

To assess the effect of mSNVs on the p53–BCL-2 PPIs, ΔΔG binding affinity changes were predicted using DDMut-PPI v1.0, based on the p53–BCL-2 complex structure 8HLL [21]. DDMut-PPI extends DDMut with a robust Siamese network architecture incorporating graph-based signatures and existing mutagenesis data. The model was enhanced with a graph neural network to capture structural and physicochemical features of PPI interfaces. BCL-2 was selected because p53 directly regulates apoptosis through transcriptional repression of *BCL2*, making alterations in this interaction biologically relevant.

#### 2.4.5. Statistical Analysis

Statistical analyses were performed using Python v3.10 (SciPy and Statsmodels libraries). Differences in *TP53* mutation frequencies between cancer types were first assessed using a Chi^2^ test of independence. Because some contingency table cells contained low expected counts, pairwise comparisons between cancer types were subsequently performed using Fisher’s exact test. Differences in the distribution of mutations between *TP53* exons 5 and 6 across cancer types were evaluated using a Chi^2^ test of independence. To assess exon-specific mutation enrichment within each cancer type, exact binomial tests were performed by comparing the number of mutations observed in exon 6 relative to exon 5. For PolyPhen-2 pathogenicity predictions, variants were analyzed at the variant level and classified as damaging (possibly damaging or probably damaging) or non-damaging (benign). Differences in pathogenicity distributions between cancer types were assessed using Fisher’s exact test. Comparisons of continuous structural stability and conformational dynamics metrics, including FoldX ΔΔG values and ENCoM-derived $\Delta \Delta S_{vib}$ values, between *TP53* exons 5 and 6 were conducted using the non-parametric Mann–Whitney U test, as these variables did not meet assumptions of normality. Two-sided *p*-values were reported. For all analyses involving multiple pairwise comparisons, *p*-values were adjusted using the Benjamini–Hochberg false discovery rate (FDR) procedure. An adjusted *p*-value < 0.05 was considered statistically significant.

## 3. Results

### 3.1. Mutation Identification

**Figure 1:**
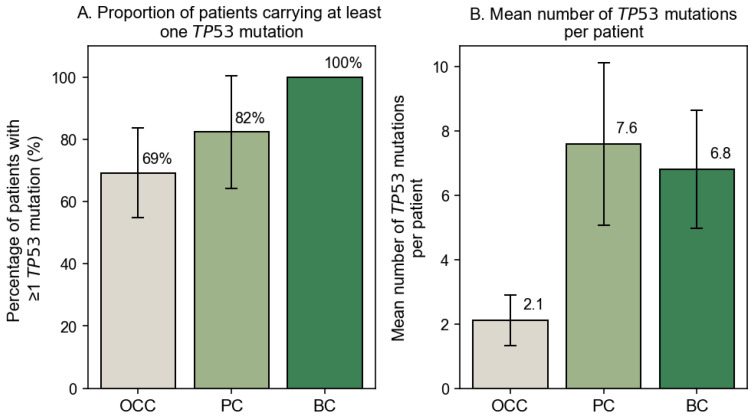
Frequency of *TP53* mutations across cancer types. (**A**) Percentage of patients carrying at least one somatic *TP53* mutation among the OCC (*n* = 39), PC (*n* = 17), and BC (*n* = 20) groups. (**B**) Mean number of somatic *TP53* mutations per patient in each cancer type. Error bars represent 95% confidence intervals.

Sequence analysis was successfully performed for all samples except one OCC and one PC specimen, which were excluded due to poor chromatogram quality and insufficient alignment confidence. The proportion of patients carrying at least one *TP53* mutation differed across cancer types, with 69.23% in OCC (*n* = 27), 82.35% in PC (*n* = 17), and 100% in BC (*n* = 20). The corresponding mutation burdens were 2.1, 7.6, and 6.8 mutations per patient, respectively (Figure 1A,B). A statistically significant association between cancer type and mutation status (χ^2^ = 8.414, *p* = 0.015) indicated that mutation frequencies are not uniformly distributed across OCC, PC, and BC. Pairwise comparisons with Fisher’s exact test followed by Benjamini–Hochberg FDR correction showed that BC harbored a significantly higher proportion of mutated cases compared with OCC (*p* = 0.014). In contrast, no statistically significant differences were observed between OCC and PC (*p* = 0.231) or between PC and BC (*p* = 0.231). These results indicate that the overall difference detected by the Chi^2^ test is primarily driven by the high mutation frequency observed in BC.

**Figure 2:**
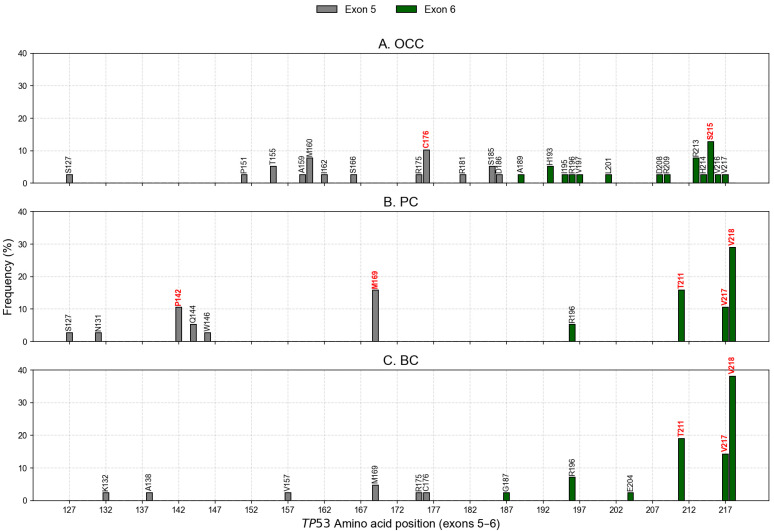
Distribution of *TP53* mSNVs across exons 5 and 6. The *X*-axis displays all amino acid positions corresponding to exons 5 (codons 126–186) and 6 (codons 187–224). Vertical markers indicate positions where somatic mutations were detected in OCC (**A**), PC (**B**), and BC (**C**). Their height represents the percentage of patients carrying a mutation at that position. Mutations highlighted in red correspond to recurrent hotspot variants with the highest frequencies within each cancer type.

Mutation profiling identified 31, 14, and 15 unique nonsynonymous variants (nsSNVs) in OCC, PC, and BC, respectively. When accounting for inter-individual recurrence, these corresponded to 39 mSNVs and one nonsense in OCC, 38 mSNVs and five nonsenses in PC, and 42 mSNVs and two nonsenses in BC. In OCC, the most recurrent variants were S215N (c.644G>A) and R213Q (c.638G>A), detected in 10.26% and 7.69% of patients, respectively (Figure 2A). PC and BC exhibited partially overlapping profiles, with V218M representing the predominant mutation in both groups (28.95% in PC and 38.10% in BC), followed by T211A (15.79% in PC and 19.05% in BC; Figure 2B,C). The uncatalogued COSMIC variant V217L was also detected at notable frequencies (10.53% in PC and 9.52% in BC). Co-occurrence analysis revealed that both V217L and V218M mutations were simultaneously detected in a subset of patients, specifically in 4 PC patients and 3 BC patients, whereas no such double-mutant cases were observed in OCC. In addition, M169T (c.506T>C) was among the most recurrent mutations in PC (15.79%). These mutational patterns and their distribution across exons 5 and 6 are illustrated in Figure 2. Notably, the codon C176 in OCC exhibited three distinct mSNVs (C176R, C176S, and C176Y), which collectively accounted for 10.26% of all mutation events in this group. Similarly, in PC, codon P142 displayed two independent substitutions (P142A and P142T), reaching a combined frequency of 10.53% (Figure 2B).

**Figure 3:**
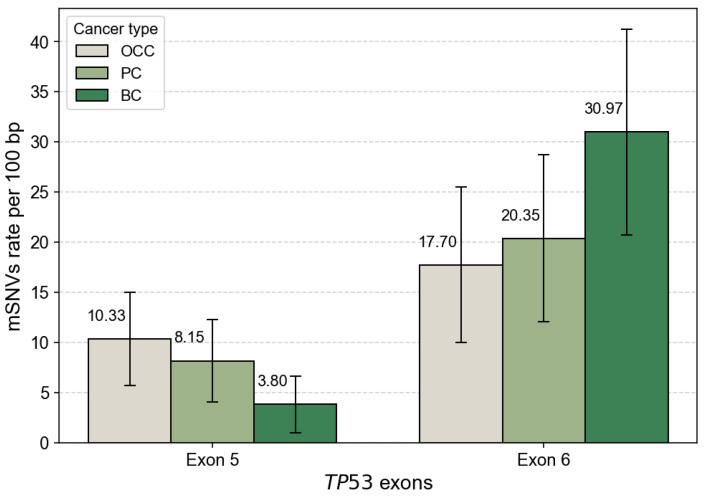
*TP53* mSNV rates normalized by exon length. Bar plots show the number of somatic *TP53* mutations per 100 bp in exon 5 and exon 6 for OCC, PC, and BC. Mutation counts were normalized by exon size (exon 5 = 184 bp, exon 6 = 113 bp) to account for length-dependent differences in mutation probability. Error bars represent 95% confidence intervals based on a Poisson approximation of mutation counts.

After normalization by exon length, mutation rates were comparable between exons 5 and 6 in OCC, with 10.33 and 17.70 mutations per 100 bp, respectively. In contrast, PC and BC displayed higher mutation densities in exon 6 than in exon 5 (Figure 3). This trend was particularly pronounced in BC, which exhibited the highest overall exon-normalized mutation rate among the three cancer types (3.80 vs. 30.97 mutations per 100 bp for exons 5 and 6, respectively).

The distribution of *TP53* mSNVs between exons 5 and 6 across cancer types was further evaluated using a Chi^2^ test of independence. This global analysis revealed a significant association between exon location and cancer type (χ^2^ = 9.822, *p* = 0.007), indicating that the relative contribution of exons 5 and 6 to the mutational burden differs among OCC, PC, and BC. After FDR adjustment, exon 6 showed a highly significant enrichment of mutations in BC (*p* = 0.00045), whereas no significant difference between exons 5 and 6 was observed in OCC (*p* = 1.000) or PC (*p* = 0.384). These results also indicate that the global exon-level difference is primarily driven by a strong enrichment of exon 6 mutations in BC, while exon-level mutation frequencies remain comparable in OCC and PC.

### 3.2. Functional Impact of mSNVs

**Figure 4:**
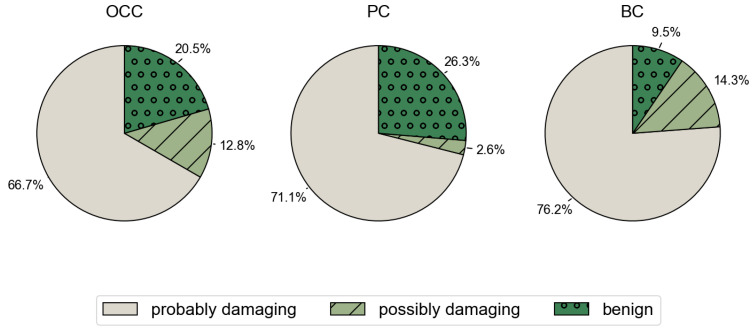
Distribution of PolyPhen-2 predicted functional impact across cancer types. Pie charts show the proportion of *TP53* mSNVs classified by PolyPhen-2 as probably damaging, possibly damaging, or benign in OCC, PC, and BC.

The analysis of functional impact using PolyPhen-2 indicated increased pathogenicity for most mutations: 66.7%, 71.1%, and 76.3% of the mSNVs were probably damaging in OCC, PC, and BC, respectively. Furthermore, the percentages of potentially damaging mutations to p53 were 12.8%, 2.6%, and 13.4%, respectively (Figure 4).

**Figure 5:**
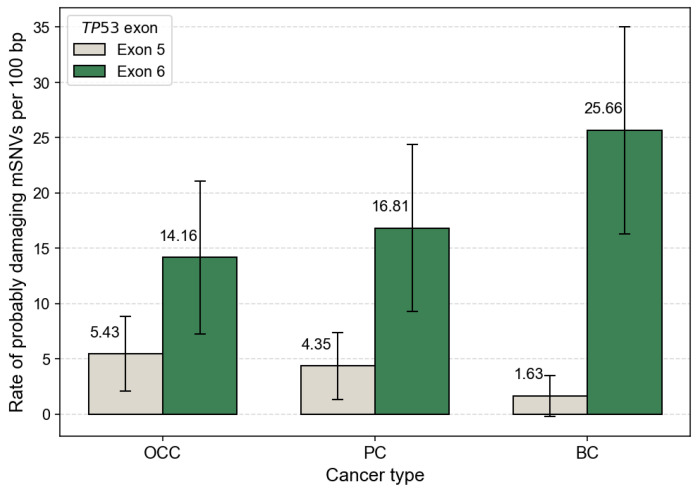
Exon-normalized rate of *TP53* mSNVs predicted as ‘probably damaging’ by PolyPhen-2. Bars represent the number of probably damaging mSNVs per 100 bp in exon 5 and exon 6 for OCC, PC, and BC. Mutation counts were normalized by exon size (exon 5 = 184 bp, exon 6 = 113 bp). Error bars indicate 95% confidence intervals based on a Poisson approximation of mutation counts.

When focusing on PolyPhen-2 predictions classified as probably damaging, exon 6 consistently showed higher mutation rates per 100 bp than exon 5 across all cancer types. This trend was observed in OCC, PC, and BC, even after normalization by exon length, indicating that the predominance of exon 6 mutations is unlikely to be explained solely by exon size (Figure 5).

However, pairwise comparisons using Fisher’s exact test did not reveal any statistically significant differences in the proportion of damaging variants between cancer types (OCC vs. PC: *p* = 0.599; OCC vs. BC: *p* = 0.324; PC vs. BC: *p* = 0.227). These results indicate that despite numerical differences, PolyPhen-2 predicts broadly comparable levels of pathogenicity across OCC, PC, and BC.

### 3.3. Impact of mSNVs on Structural Stability

**Figure 6:**
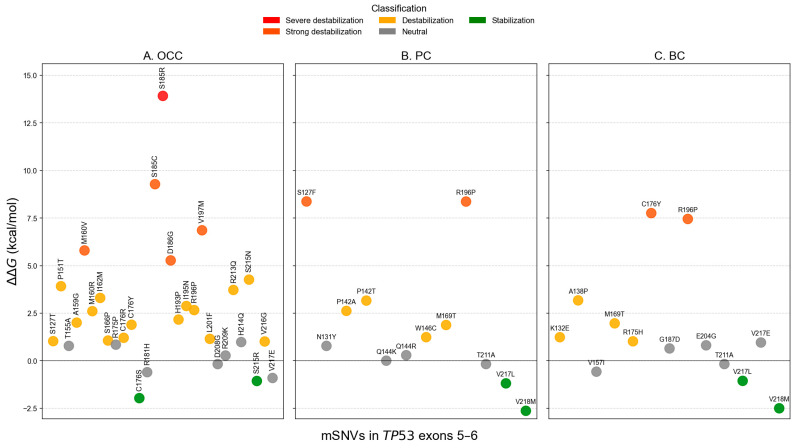
Structural stability impact of *TP53* mSNVs predicted by FoldX across cancer types. Scatter plots show ΔΔG values (kcal/mol) for mSNVs identified in OCC (**A**), PC (**B**), and BC (**C**). Each point represents a single variant, colored according to stability classification: stabilization (ΔΔG ≤ −1 kcal/mol), neutral (−1 < ΔΔG ≤ 1 kcal/mol)), destabilization (1 < ΔΔG ≤ 5 kcal/mol)), strong destabilization (5 < ΔΔG ≤ 10 kcal/mol)), and severe destabilization (ΔΔG > 10 kcal/mol)).

FoldX ΔΔG analysis identified two stabilizing variants in OCC (C176S and S215R), representing 5.13% of all mutations (Figure 6A). Twenty variants (66.67%) were predicted to be destabilizing, including one severely destabilizing mutation, S185R, with a ΔΔG of 13.915 kcal/mol. In PC and BC, two variants were predicted to stabilize the protein: V217L and V218M (Figure 6B,C). These stabilizing variants accounted for 39.47% and 47.62% of mSNVs in PC and BC, respectively. Six destabilizing variants were also detected in each cancer type: S127F, P142A, P142T, W146C, M169T, and R196P in PC; and K132E, A138P, M169T, R175H, C176Y, and R196P in BC. The unregistered COSMIC mSNV T211A exhibited a ΔΔG value within the neutral range and therefore was not classified as stabilizing.

**Figure 7:**
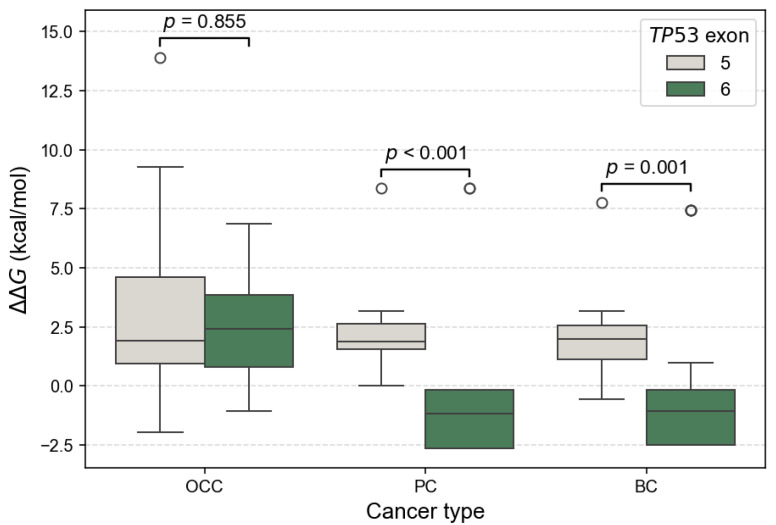
Comparison of FoldX ΔΔG distributions between *TP53* exons 5 and 6 across cancer types. Boxplots show the distribution of ΔΔG values for mSNVs stratified by OCC, PC, and BC. Higher ΔΔG values indicate stronger destabilizing effects on p53 stability. Statistical differences between their ΔΔG distributions were assessed using two-sided Mann–Whitney U tests.

Additionally, the distribution of ΔΔG values by exon showed exon and cancer-type-dependent patterns. It revealed consistently higher ΔΔG for exon 5 variants across the three cancer types (Figure 7). In contrast, exon 6 variants showed a marked reduction in ΔΔG in PC and BC. Differences in mutation-induced structural stability between *TP53* exons 5 and 6 were assessed using the non-parametric Mann–Whitney U test. FoldX ΔΔG distributions revealed no significant difference between exon 5 and exon 6 variants in OCC (*p* = 0.855), indicating comparable destabilizing effects on p53 structure in this cancer type. In contrast, exon 6 mutations were significantly more stabilizing than exon 5 mutations in PC (*p* = 1.22 × 10^−5^) and BC (*p* = 0.0013), as reflected by lower and predominantly negative ΔΔG values. These results indicate a cancer-type dependent effect of exon location, with exon 6 variants exerting a stabilizing influence on p53 structure in PC and BC.

### 3.4. Impact of mSNVs on Thermodynamic Stability

**Figure 8:**
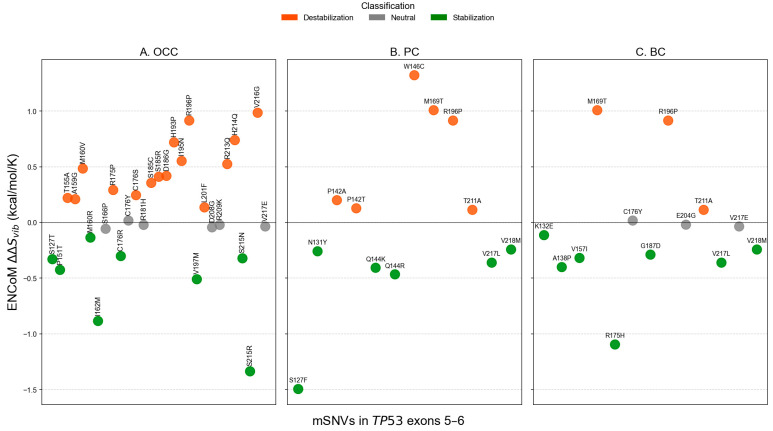
ENCoM-derived $\Delta \Delta S_{vib}$ conformational dynamics predictions for *TP53* mSNVs across cancer types. Scatter plots show $\Delta \Delta S_{vib}$ values calculated using the ENCoM elastic network model for *TP53* mSNVs identified in OCC (**A**), PC (**B**), and BC (**C**). Positive $\Delta \Delta S_{vib}$ values indicate increased molecular flexibility (destabilizing effect), whereas negative values indicate decreased flexibility (stabilizing effect). Each point represents one mSNV, colored according to its predicted impact on protein dynamics: stabilizing ($\Delta \Delta S_{vib}$< −0.1 kcal·mol^−1^·K^−1^), neutral), neutral (−0.1 ≤ $\Delta \Delta S_{vib}$ ≤ +0.1 kcal·mol^−1^·K^−1^), and destabilizing ($\Delta \Delta S_{vib}$> +0.1 kcal·mol^−1^·K^−1^).

Analysis of conformational dynamics using ENCoM revealed distinct flexibility patterns across cancer types. In OCC, most mSNVs increased the vibrational entropy of the DBD, indicating enhanced local flexibility (16 flexible vs. 8 rigidifying variants, Figure 8A). In contrast, PC and BC displayed the opposite trend, with a higher proportion of mSNVs predicted to decrease vibrational entropy, consistent with increased structural rigidity of the DBD. Variants showing the strongest flexibilizing effects included W146C in PC and M169T in both PC and BC, which exhibited the largest positive $\Delta \Delta S_{vib}$ values (Figure 8B,C). Conversely, mutations such as S215R ($\Delta \Delta S_{vib}$ = −1.337 kcal/mol/K) in OCC, S127F ($\Delta \Delta S_{vib}$ = −1.496 kcal/mol/K) in PC, and R175H ($\Delta \Delta S_{vib}$ = −1.096 kcal/mol/K) in BC demonstrated marked rigidifying. These results highlight cancer-specific differences in how *TP53* mSNVs alter the dynamic behavior of the DBD.

**Figure 9:**
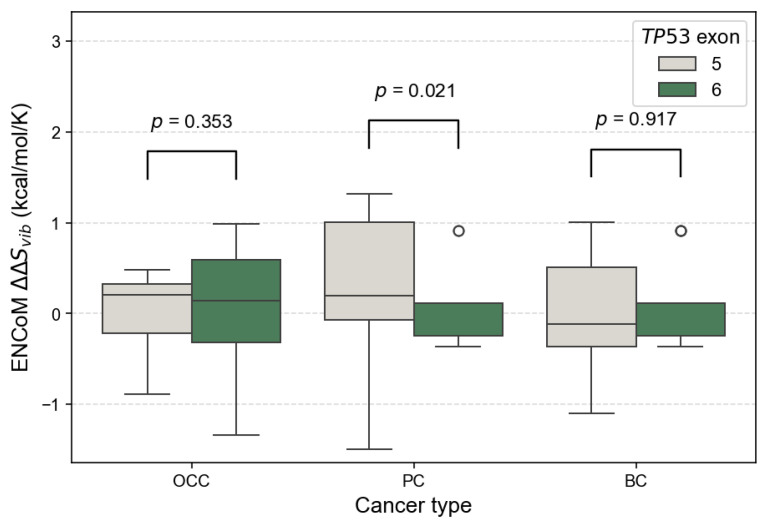
Comparison of ENCoM $\Delta \Delta S_{vib}$ distributions between *TP53* exons 5 and 6 across cancer types. Boxplots show the distribution of ENCoM $\Delta \Delta S_{vib}$ values for mSNVs stratified by OCC, PC, and BC. Higher $\Delta \Delta S_{vib}$ values indicate stronger destabilizing effects on p53 stability. Statistical differences between their $\Delta \Delta S_{vib}$ distributions were assessed using two-sided Mann–Whitney U tests.

At the exon level, the distribution of ENCoM $\Delta \Delta S_{vib}$ showed small exon type patterns. In OCC, $\Delta \Delta S_{vib}$ values were globally centered around zero for both exon 5 and exon 6 variants, indicating comparable flexibility changes, with no significant difference between exons (*p* = 0.353, Figure 9). In PC, exon 5 variants displayed higher and more dispersed $\Delta \Delta S_{vib}$ values compared to exon 6 variants, reflecting increased mutation-induced flexibility for exon 5; this difference was statistically significant (*p* = 0.021). In BC, $\Delta \Delta S_{vib}$ values were predominantly slightly negative for both exons, suggesting similar and modest rigidity changes, with no significant exon-related difference (*p* = 0.917). Overall, PC exhibited the largest contrast in $\Delta \Delta S_{vib}$ between exons, whereas OCC and BC showed relatively homogeneous flexibility profiles across exons.

### 3.5. Impact of mSNVs on p53-BCL2 Protein–Protein Interactions

**Figure 10:**
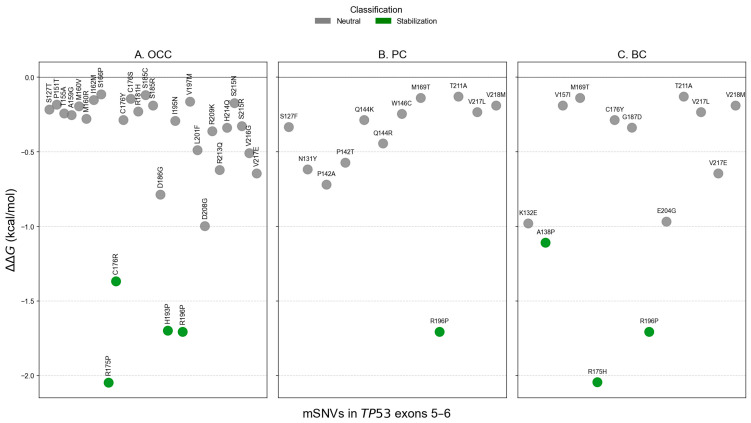
Predicted impact of *TP53* mSNVs on the p53–BCL-2 interaction across all cancer types. Scatter plots show ΔΔG values (kcal/mol) for the p53–BCL-2 complex for *TP53* mSNVs identified in OCC (**A**), PC (**B**), and BC (**C**). Negative ΔΔG values indicate a strengthening of the p53–BCL-2 interaction (stabilization of binding), whereas values close to zero correspond to a neutral effect. Variants were classified as stabilizing (ΔΔG ≤ −1 kcal/mol) or neutral (−1 < ΔΔG < 1 kcal/mol). No mutation reached the destabilizing range (ΔΔG ≥ 1 kcal/mol) in this dataset.

Analysis of p53–BCL-2 binding affinity showed that none of the *TP53* mSNVs identified in OCC, PC, or BC had a destabilizing effect on this interaction. Most variants exhibited either minimal impact or increased binding affinity (ΔΔG ≤ −1 kcal/mol), with several cancer-specific stabilizing mutations detected. In OCC, the stabilizing mutations included R175P, C176R, H193P, and R196P, whereas PC and BC shared the stabilizing variant R196P (Figure 10A–C). Additional stabilizing effects were observed for A138P and R175H in BC. Notably, two of the strongest affinity-enhancing effects were found at codon 175, with R175P in OCC (ΔΔG = −2.048 kcal/mol) and R175H in BC (ΔΔG = −2.045 kcal/mol), suggesting that alterations at this hotspot may consistently reinforce p53–BCL-2 binding across cancer types.

Detailed variant annotations and computational predictions are provided in the Supplementary Materials.

## 4. Discussion

A significant variation in *TP53* mutation frequency across cancer types was observed, as demonstrated by the Chi^2^ analysis (*p* = 0.015), with BC exhibiting the highest proportion of mutated cases. Pairwise comparisons further indicated that this difference was primarily driven by BC, whereas mutation frequencies in OCC and PC were not significantly different after correction for multiple testing. The elevated mutational burden observed in BC may reflect cancer-type specific biological and etiological factors. Mammary epithelial cells are subject to intense hormonal regulation and metabolic activity, particularly in estrogen-responsive contexts, which has been associated with increased oxidative stress and the generation of reactive oxygen species capable of inducing DNA damage [22]. Previous studies have also reported a correlation between oxidative stress levels and BC aggressiveness, suggesting sustained pressure on DNA damage response pathways such as p53 [23]. In contrast, PC progression is largely driven by androgen receptor signaling, which may impose distinct selective pressures on *TP53* compared with hormonally regulated breast tissue [24]. OCC, on the other hand, is predominantly associated with environmental carcinogens such as tobacco, alcohol, and viral infections, particularly HPV, where *TP53* alterations are present but not uniformly required for tumor development [25]. Consistently, OCC samples in this study displayed the lowest average mutational burden per patient. Despite this low burden, *TP53* mutations in OCC were characterized by broad codon distribution and low recurrence, indicative of a heterogeneous mutational landscape.

Across cancer types, the spectrum of *TP53* variants differed in both composition and diversity. In OCC, we identified 39 mSNVs, one nonsense mutation, and two small indels. In contrast, PC harbored 38 mSNVs and five nonsense mutations, while BC presented 42 mSNVs and two nonsense mutations, with no indels detected in either PC or BC. Although the overall proportions of variant classes were broadly comparable, notable differences emerged in the diversity and distribution of unique variants across exons. At the variant level, OCC exhibited approximately twice as many unique nsSNVs as PC and BC (31 versus 14 and 15, respectively), despite a lower average number of mutations per patient. This pattern suggests a broader mutational heterogeneity in OCC, potentially reflecting diverse mutagenic exposures rather than recurrent hotspot-driven alterations. The presence of indels exclusively in OCC further supports this interpretation, as such variants are often associated with error-prone DNA repair mechanisms induced by environmental carcinogens. These observations are consistent with previous large-scale analyses of *TP53* mutational landscapes. In particular, Mroz et al. reported increased heterogeneity and a reduced recurrence of *TP53* mutations in head and neck cancers compared with other solid tumors based on The Cancer Genome Atlas (TCGA) data [26]. Together, these findings suggest that *TP53* alterations in OCC may arise from a wider range of mutational processes, whereas PC and BC appear to be characterized by more recurrent and structurally constrained mutation patterns.

Our analyses further revealed significant exon-dependent distribution of *TP53* mSNVs across cancer types. At the global level, exon location was significantly associated with cancer type (*p* = 0.007), with exon 6 showing a marked enrichment of mutations in BC (*p* < 0.001), whereas no significant exon-specific difference was observed in OCC or PC. This enrichment of exon 6 mutations in BC contrasts with several large-scale studies reporting a predominance of exon 5 mutations within the *TP53* DBD across diverse populations. For instance, Leroy et al. (2014) described exon 5 as one of the most frequently mutated regions in multiple cancer types [27]. Similarly, in a Chinese cohort of 50 patients with BC, Lu et al. (2023) reported that exon 5 harbored the highest proportion of *TP53* mutations (24.14%), while exon 6 was among the least frequently mutated regions [28].

However, studies providing detailed exon level *TP53* mutation frequencies in African populations remain scarce, limiting direct comparisons and the identification of robust population wide trends. In this context, population focused studies suggest substantial regional variability. For example, Ouedraogo et al. (2022) reported that all *TP53* mutations identified in a cohort of 133 BC patients from Burkina Faso were located in exon 4, further illustrating the pronounced heterogeneity of *TP53* mutation distributions across African populations [29]. Taken together, these findings indicate that *TP53* exon level mutation distributions are better interpreted as population dependent statistical tendencies rather than universal mutational patterns. In this context, our results suggest that selective pressures acting on the *TP53* DBD may differ across tumor and population contexts, with exon 6 playing a particularly prominent role in BC within our cohort.

Notably, exon 6 in the PC and BC cohorts also harbored recurrent truncating mutations, including Y220* and E221*. These nonsense mutations are expected to severely compromise the structural integrity of the p53 DBD as they result in premature termination within a region critical for proper folding, zinc coordination, and DBD activity [30]. Although PolyPhen-2 predictions did not reveal statistically significant differences in pathogenicity proportions across cancer types, the combined enrichment of exon 6 mutations and the presence of truncating variants support the notion that alterations affecting this exon may exert substantial functional consequences. Jointly, these findings suggest that exon 6 mutations may contribute to BC development through mechanisms that extend beyond mutation frequency alone.

At the structural and dynamic levels, *TP53* mutations identified in PC and BC displayed relatively moderate average effects when considered globally. However, a clear exon-dependent divergence emerged upon stratification. Exon 6 variants were significantly more stabilizing than exon 5 variants in PC (*p* < 0.001) and BC (*p* = 0.001), whereas no exon-dependent difference was observed in OCC (*p* = 0.855). In parallel, ENCoM-based $\Delta \Delta S_{vib}$ analysis uncovered a partially independent pattern: exon 5 mutations in PC induced significantly greater flexibility changes than exon 6 mutations (*p* = 0.021), while no significant exon-specific differences in conformational dynamics were detected in OCC (*p* = 0.353) or BC (*p* = 0.917). Despite the greater mutational diversity observed in OCC, *TP53* mutations in this cancer type and exon 5 mutations across all three cancers were predominantly destabilizing. Such destabilizing alterations are frequently associated with loss-of-function (LOF) effects, as they impair the structural integrity of the p53 DNA-binding domain, leading to reduced protein stability, defective DNA binding, and compromised transcriptional activity [30]. Consistent with the exposure-related etiology of OCC, *TP53* mutations in this context likely arise from multiple mutational processes and exert variable, often deleterious, effects on p53 stability and conformational dynamics.

In particular, the exon 6-associated stabilization observed in this study was largely driven by recurrent mSNVs such as V217L and V218M, which were shared between the PC and BC cohorts. Although direct functional data on V217L remains scarce and this variant is not yet catalogued in major cancer mutation databases such as COSMIC, its recurrent detection and stabilizing effect in our cohort suggest potential biological relevance. Similarly, V218M is a rare *TP53* variant with limited functional characterization to date. Notably, Jena et al. recently identified V218M as a novel *TP53* mutation in acute erythroid leukemia and suggested that it may impair p53 transcriptional activity and contribute to aggressive disease behavior [31]. Accumulation of mutant p53 proteins is known to profoundly alter transcriptional regulation, including impaired activation of canonical tumor suppressor targets such as CDKN1A, BAX, and PUMA, as well as dysregulated control of non-canonical genes involved in proliferation, survival, and metastasis. Although MDM2 primarily interacts with the N-terminal domain of p53, stabilizing mutations within the DNA-binding domain can indirectly promote mutant p53 accumulation by modulating ubiquitination and proteasomal degradation pathways. Supporting this mechanism, Liu et al. (2025) demonstrated that certain *TP53* mutations enhance mutant p53 stability through altered ubiquitination, leading to increased oncogenic phenotypes in cellular, organoid, and animal models [32]. More broadly, stabilizing mutations of p53 have been increasingly linked to the accumulation of mutant protein and the acquisition of oncogenic GOF properties rather than the restoration of tumor-suppressive activity [33]. In this context, the recurrent detection of stabilizing V217L and V218M mutations supports the hypothesis that selective pressures acting on exon 6 favor structurally stable mutant p53 variants in PC and BC within the cohort.

Beyond their effects on p53 structural stability and conformational dynamics, *TP53* mutations may also influence tumor progression through altered PPIs. All analyzed mSNVs were predicted to stabilize the interaction between p53 and BCL-2, suggesting a general tendency toward enhanced binding affinity in the mutant context. This effect was particularly pronounced for specific variants, including R175P, H193P, R175H, and R196P. Although most of these mutant residues were not located in the PPI interface, the predicted stabilization of the p53–BCL-2 complex may have important functional implications. Increased association with BCL-2 could impair the pro-apoptotic functions of p53 by reinforcing anti-apoptotic signaling. In particular, recurrent mutations such as R175H and R196P have been extensively characterized as oncogenic *TP53* variants and are frequently associated with loss of canonical p53 transcriptional activity as well as GOF properties [2,33,34]. The observation that both cancer-specific and shared *TP53* mutations enhance p53–BCL-2 interaction supports a model in which mutant p53 contributes to tumor cell survival by promoting resistance to apoptosis. In this context, stabilization of the p53–BCL-2 complex may represent an additional mechanism through which mutant p53 exerts oncogenic effects, complementing structural stabilization and altered conformational dynamics. Such effects may be particularly relevant in cancers where apoptotic escape constitutes a key driver of disease progression and therapeutic resistance.

### 4.1. Limitations

This study has some limitations. First, the retrospective design including the absence of associated clinical, pathological information and the restriction to exons 5 and 6 of *TP53* limit the generalization of the findings to the full mutational spectrum of the gene. Second, the analyses were based exclusively on in silico predictions, and experimental validation of the structural, functional, dynamic, and PPI effects were not performed. In addition, the absence of matched normal samples precluded direct discrimination between somatic and rare germline variants. Despite these limitations, the integrative computational approach provides valuable insights into *TP53* mutational patterns in an underrepresented population and establishes a framework for future experimental and large-scale genomic studies.

### 4.2. Medical and Clinical Implications

From a medical perspective, the enrichment of recurrent exon 6 *TP53* mutations in PC and BC, combined with their stabilizing effects on mutant p53 and enhanced interaction with the anti-apoptotic protein BCL-2, suggests a potential contribution to tumor progression through GOF mechanisms. Such stabilized mutant p53 proteins have been associated with increased tumor aggressiveness, resistance to apoptosis, and poor clinical outcomes. In contrast, the predominance of destabilizing *TP53* mutations observed in OCC, particularly in exon 5, likely reflects LOF alterations leading to impaired DNA damage response and genomic instability, consistent with exposure-driven tumorigenesis. These differences highlight the importance of exon-specific *TP53* profiling, as distinct mutation classes may underlie divergent biological behaviors and therapeutic vulnerabilities across cancer types. Although clinical validation is required, these findings support the potential utility of exon-level *TP53* profiling for improved biological stratification of cancers.

## 5. Conclusions

This study provides an exon-specific functional and structural analysis of *TP53* mutations in OCC, PC, and BC from a Senegalese cohort. Our results highlight distinct cancer- and exon-dependent mutation patterns, with exon 6 mutations in PC and BC showing stabilizing effects suggestive of gain-of-function properties, in contrast to predominantly destabilizing mutations in OCC. Overall, this work underscores the importance of exon-level analyses and population specific data for understanding the biological impact of *TP53* alterations in cancer.

## Data Availability

The original contributions presented in this study are included in the article/Supplementary Materials. Further inquiries can be directed to the corresponding author.

## Supplementary Materials

The following supporting information can be downloaded at: https://www.mdpi.com/article/10.3390/genes17020245/s1, Table S1: Complete list of *TP53* variants identified in exons 5 and 6 in OCC with Pphen-2, ΔΔG, $\Delta \Delta S_{vib}$, BCL-2 affinity results; Table S2: Complete list of *TP53* variants identified in exons 5 and 6 in PC with Pphen-2, ΔΔG, $\Delta \Delta S_{vib}$, BCL-2 affinity results; Table S3: Complete list of *TP53* variants identified in exons 5 and 6 in BC with Pphen-2, ΔΔG, $\Delta \Delta S_{vib}$, BCL-2 affinity results.

## Abbreviations

The following abbreviations are used in this manuscript:

BC	Breast Cancer
DBD	DNA Binding Domain
ENCoM	Elastic Network Contact Model
FDR	False Discovery Rate
GOF	Gain-Of-Function
HALD	Hôpital Aristide Le Dantec
LOF	Loss-Of-Function
mSNVs	Missense Single Nucleotide Variants
NM	Numerical Mutation
NMA	Normal Mode Analysis
OCC	Oral Cavity Cancer
PC	Prostate Cancer
PPIs	Protein–Protein interactions
*TP53*	Tumor Protein 53
UCAD	Université Cheikh Anta Diop
ΔG	Gibbs Free Energy
ΔΔG	Gibbs Free Energy Difference
$\Delta S_{vib}$	Vibrational Entropy
$\Delta \Delta S_{vib}$	Vibrational Entropy Difference
